# Costs of HIV prevention services provided by community-based organizations to female sex workers in Nigeria

**DOI:** 10.1371/journal.pone.0282826

**Published:** 2023-03-13

**Authors:** Nerissa Nance, Andrea Salas-Ortiz, Kayode Ogungbemi, David Akeju, Adejumoke G. Oluwayinka, Idoteyin Ezirim, James Anenih, Ogbonna Amanze, Godpower Omoregie, Sani H. Aliyu, Sergio Bautista-Arredondo

**Affiliations:** 1 National Institute of Public Health, Mexico (INSP), Cuernavaca, Mexico; 2 University of California, Berkeley, Berkeley, CA, United States of America; 3 Centre for Health Economics, University of York, York, United Kingdom; 4 National Agency for the Control of AIDS (NACA), Abuja, Nigeria; 5 University of Lagos (UNILAG), Lagos, Nigeria; 6 Society for Family Health (SFH), Abuja, Nigeria; University of the Witwatersrand, SOUTH AFRICA

## Abstract

**Background:**

Nigeria has been consistently targeted in sub-Saharan Africa as an HIV-priority country. Its main mode of transmission is heterosexual, and consequently, a key population of interest is female sex workers (FSWs). While HIV prevention services are increasingly implemented by community-based organizations (CBOs) in Nigeria, there is a paucity of evidence on the implementation costs of these organizations. This study seeks to fill this gap by providing new evidence about service delivery unit cost for HIV education (HIVE), HIV counseling and testing (HCT), and sexually transmitted infection (STI) referral services.

**Methods:**

In a sample of 31 CBOs across Nigeria, we calculated the costs of HIV prevention services for FSWs taking a provider-based perspective. We collected 2016 fiscal year data on tablet computers during a central data training in Abuja, Nigeria, in August 2017. Data collection was part of a cluster-randomized trial examining the effects of management practices in CBOs on HIV prevention service delivery. Staff costs, recurrent inputs, utilities, and training costs were aggregated and allocated to each intervention to produce total cost calculations, and then divided by the number of FSWs served to produce unit costs. Where costs were shared across interventions, a weight proportional to intervention outputs was applied. All cost data were converted to US dollars using the mid-year 2016 exchange rate. We also explored the cost variation across the CBOs, particularly the roles of service scale, geographic location, and time.

**Results:**

The average annual number of services provided per CBO was 11,294 for HIVE, 3,326 for HCT, and 473 for STI referrals. The unit cost per FSW tested for HIV was 22 USD, the unit cost per FSW reached with HIV education services was 19 USD, and the unit cost per FSW reached by STI referrals was 3 USD. We found heterogeneity in total and unit costs across CBOs and geographic location. Results from the regression models show that total cost and service scale were positively correlated, while unit costs and scale were consistently negatively correlated; this indicates the presence of economies of scale. By increasing the annual number of services by 100 percent, the unit cost decreases by 50 percent for HIVE, 40 percent for HCT, and 10 percent for STI. There was also evidence that indicates that the level of service provision was not constant over time across the fiscal year. We also found unit costs and management to be negatively correlated, though results were not statistically significant.

**Conclusions:**

Estimates for HCT services are relatively similar to previous studies. There is substantial variation in unit costs across facilities, and evidence of a negative relationship between unit costs and scale for all services. This is one of the few studies to measure HIV prevention service delivery costs to female sex workers through CBOs. Furthermore, this study also looked at the relationship between costs and management practices—the first of its kind to do so in Nigeria. Results can be leveraged to strategically plan for future service delivery across similar settings.

## Introduction

Despite significant progress in the fight to end the global HIV epidemic, plans have yet to meet expectations; there were 1.7 million new HIV infections globally in 2019 alone [1]. The recent UNAIDS global update is a call to action for countries and multilateral organizations to increase investment in the HIV response [2]. Funding for HIV programs has experienced a global downturn over recent years, and while funding increased in 2017, this trend that is not expected to continue [3]. Moreover, there is a significant gap between funding needs and availability [3]. Where resources are stretched thin, it is imperative that they be allocated judiciously and efficiently.

Funding is particularly imperative for the sub-Saharan Africa region (which accounts for a majority of current and new infections worldwide [4]), and among key populations (which account for a majority of new infections [5]). Key populations are at particularly high risk for HIV infection, including men who have sex with men (MSM), persons who inject drugs (PWID), and FSWs [6]. In sub-Saharan Africa, the Nigerian epidemic is an example of a simultaneously generalized and concentrated epidemic. The country has the second largest epidemic in the world, with a large proportion of new infections concentrated in groups such as FSWs whose HIV prevalence is 14.4 percent [7]. The HIV prevalence in the general population is 1.4 percent [8, 9].

As part of the response to address the epidemic in these key populations, relevant government agencies and implementing partners have implemented the Minimum Prevention Package Intervention (MPPI) since 2010 [10]. The MPPI addresses HIV prevention through a multifaceted combination of behavioral, biomedical, and structural interventions [11]. While it has been integral to the HIV response in the country, there has yet to be reliable data published on the costs of the program. Understanding service costs is integral to program planning and evaluation, both nationally and internationally. In Nigeria, national governing entities have increased funding to HIV by 30 percent between 2010 and 2018, making the subject of particular national interest [12]. In particular, as HIV services transition to highly targeted and more personalized services like differentiated care [13], understanding the efficiency of CBOs-provided services for key populations will be crucial for service expansion in the coming years.

There is a considerable body of research measuring HIV transmission prevention service costs among key populations [14–26]. However, most of these studies have been focused on the case of India or have used top-down and financial costing approaches, which limits the scope and accuracy of the estimates. Moreover, very few studies analyze efficiency beyond the estimation of the unit cost of services. Our study produces novel evidence about the costs of HIV prevention services by harnessing bottom-up micro-costing data from a cluster-randomized control trial (C-RCT) of prevention services delivered to FSWs by CBOs in Nigeria. Through this analysis, we will: (1) estimate the total and unit costs of three fundamental services for FSW in Nigeria—HIV education, HIV testing, and counseling and STI treatment referrals—delivered by CBOs in 14 states of Nigeria; and (2) analyze the heterogeneity in unit costs across CBOs and assess supply-side factors linked to cost variation, such as scale and management practices.

## Methods

### Program description

The two projects that operationalized the MPPI in Nigeria in 2016 were funded by the Global Fund (GF) and USAID which financed the Strengthening HIV Prevention Services for Most-at-Risk Populations (SHiPS for MARPs) program. Both programs focused on most at-risk populations largely targeting FSWs and MSM. For our study, we focused exclusively on the FSW population that received HIV education (HIVE), HIV counseling and testing (HCT), and sexually transmitted infection (STI) referral MPPI interventions through the GF and SHiPS programs.

According to MPPI guidelines, HIVE is a behavioral intervention consisting of peer educators from the community building rapport with other FSWs and promoting a supportive network of fellows; through this network, the peers provide information on HIV prevention and the availability of health services. In addition to information, the peers distribute male and female condoms and lubricants and help facilitate referrals for biomedical interventions. Guidelines suggest that each peer educator facilitate a maximum of 12 peer sessions every six months (recommended two per month) and that each session has between eight to 15 FSWs attending a session. HCT is a biomedical intervention that consists of providing counseling with a peer counselor and HIV testing. The service delivery may be provided through site-based clinics or outreach services. STI referral is also a biomedical intervention. The MPPI required CBOs to follow the National guidelines by screening FSWs for symptoms at least quarterly and referring them for treatment as appropriate [11]. Importantly, due in large part to the challenges reaching such marginalized groups, both GF and SHiPS programs focused exclusively on supporting and strengthening community CBOs already serving these populations.

### Study sample and data collection

The methodology for sample selection is described in further detail elsewhere [27]. In summary, the costing component of this study was couched in a larger C-RCT to look at the effect of management practices on service delivery efficiency. To be included in the sample, CBOs needed to: (1) be funded by the USAID or GF programs, and (2) serve FSWs. Of the 32 sites (CBOs) initially eligible, one was later excluded because it stopped serving FSWs.

We implemented a bottom-up micro-costing approach by selecting 62 participants (two from each of the 31 CBOs) and inviting them to a data collection training. The trainees received one electronic tablet per CBO with *Qualtrics Survey Software* [28] installed. During the training, data were collected through the online survey platform. Specifically, participants learned how to collect basic facility information such as the maturity of the CBO and the total number of projects implemented by the CBO. The instrument also collected data about monthly recurrent supplies used (condoms, test kits, lubricants, non-monetary incentives for FSW, etc.), details of staff (managerial staff and volunteers) and their salaries, monthly rent and utilities expenses (electricity, internet, phone, etc.), participation in training activities, and monthly services provided (number of FSWs served by the interventions). A detailed description of the inputs included is in S1 Table. The participants subsequently collected and uploaded retrospective data for the period January 2016 to July 2017 onto the survey platform. Data from the 2016 financial year were used in the current cost calculation.

In addition to measurements for the cost calculation, we collected data on management practices through a short survey. This survey is an adaptation of a validated tool for the identification and measurement of management practices developed by Bloom, Sadun, and Van Reenen [29]. Originally, this survey included questions about the facility’s spatial and systems organization; financial management, the use of incentives and sanctions among the personnel; and establishing goals and targets. We used this survey as a template but added some additional relevant questions based on our thematic interests and knowledge of the study context, such as management dimensions related to CBO’s involvement with the community; CBO autonomy; CBO managers’ level of satisfaction with their job, and retention of talent (S2 Table).

The survey asked participants a series of questions on a Likert scale. The answers took values from 1 to 5, where lower values were related to negative answers (never true, never, strongly disagree, or very dissatisfied) and higher values to positive responses (always true, always, strongly agree, or extremely satisfied). The responses also considered the option “does not apply”, which was recorded as 0.

### Data analysis

#### Cost calculation and cost heterogeneity

We used micro-costing to estimate economic costs from the perspective of service providers comparable across CBOs. This is a bottom-up and direct costing method. For our study, costs, prices, and quantities for essential input (collected from primary sources, mainly electronic and paper records at the facility level) were multiplied and summed up across each input category (S1 Table). Total cost of each service was calculated as follows:

$${Total\;Cost}_{ik}=\sum_{j=1}^{4}{CI}_{jik}$$ (1)

Where *Total Cost*_*ik*_ denotes the annual total cost of intervention *i* (1 = HCT, 2 = HIVE, 3 = STI) at the CBO *k*. *CI* denotes the total annual cost of input *j* (1 = recurrent supplies, 2 = personnel salaries (including staff and volunteers), 3 = rent and utilities, and 4 = training-related costs), calculated by multiplying each input by its price. The cost of the volunteers was calculated by multiplying the number of volunteers that worked in the month by their stipends and summed over the year. Notably, the remuneration per volunteer might change depending on their outputs (number of educational sessions given for HIVE and number of people tested for HCT). Volunteers were considered implementing staff and worked only on one intervention, thus the cost of volunteers that worked on the same interventions was assigned to that specific service (S1 Table). However, inputs that were used across all interventions (i.e., some recurrent supplies, managerial staff, rent and utilities, and training activities) were summed and assigned to intervention *i* through an output-based weight. These weights were calculated as the number of people reached by intervention *i* in CBO k divided by the total number of people reached by any intervention in k. This is expressed as:

$$W_{i}=number\;of\;people\;reached_{ik}/total\;number\;reached_{k}$$ (2)

Since the provision of STI referrals does not require recurrent supplies, no costs were allocated to this intervention for this category. For this allocation, an extended version of the output-based weights were created following Eq 2, but excluding STI outputs. It is also worth noting that these costs did not include any overhead, start-up costs, or donated goods, and that HIVE intervention included special recurrent supplies in the form of non-monetary incentives that were used during the HIV education sessions. Cost of inputs varied across CBOs, however, the cost of some recurrent supplies such as HIV test kits, condoms, and lubricants were provided by the program managers, since they bought these inputs. The non-monetary incentives used for HIVE were bought by the volunteers, thus, we valued these items at local market prices. Further information about input allocation to each intervention is found in S1 Table. The unit cost for each intervention was subsequently calculated as follows:

$$Unit\;cost_{ik}=total\;cost_{ik}/Total\;number\;of\;services\;provided_{ik}$$ (3)

All cost data were converted to USD dollars using the 2016 Nigerian Central Bank average mid-year exchange rate. The exchange rate considered was 252.74 NGN per one USD dollar.

To better understand the extent of the heterogeneity of the cost data, we examined it in several ways. We first looked at costs by geographic region and compared them to the HIV prevalence of the region. We then looked more closely at the breakdown of each intervention’s cost by their inputs. We decomposed the total cost by calculating the proportion of input cost by input category and then we averaged those proportions across all CBOs. Finally, we examined the outputs of each intervention over time to assess differences in service provision throughout the year. Since we collected data retrospectively, some of the monthly data overlapped, i.e., information from one month was reported in the following month. To account for this, we calculated the rolling 3-month average number of services for each intervention as:

$$q_{im}=\left(\frac{1}{3}\right)[q_{im-1}+q_{im}+q_{im+1}]$$ (4)

Where *q* is the number of services provided by intervention *i* in month *m*.

#### Cost variation by service scale

Given previous evidence about the role of service scale (annual number of services produced or patients served) in the variation of costs [13, 28], we tested the relationship between unit costs and service scale controlling for funding program using ordinary least squares (OLS) log-log regression model. We specified two models. In the first, we regressed costs on service scale only. In our second model, we regressed costs with scale and funding program. The general model is represented as follows:

$$Cost_{ik}=a_{ik}+b_{1}S_{ik}+b_{2}C_{k}+e_{ik}$$ (5)

In Eq (1), *Cost*_*ik*_ depicts the unit cost of providing an intervention *I* (1 = HCT, 2 = HIVE, 3 = STI) at the CBO *k*. *a* is a constant and for analysis represents the average fixed cost of implementing the provision of service *i* in CBO *k*. *S* represents the level of service scale (total number of services provided) and *C* represents the funding agency of the program (1 if the CBO belonged to the SHiPS program, 0 otherwise). Finally, *e*_*ik*_ is an unobserved error term that captures the random variation in the costs. Given the sample size, we decided not to include additional covariates such as management practices in the model.

#### Management practices

Finally, we explored the different management practices at the CBOs and their relationship to costs. Data on management practices were analyzed by summing the Likert scale questions for each category of management. There were 13 categories, with five items on average; positive responses had higher scores. These categories were: workplace spatial organization, workplace systems organization, financial management, incentives, sanctions, goals/targets, retention of talent, external supervision, internal supervision, transparency/accountability, job satisfaction, community involvement, and perceived autonomy. An overall or general management score was calculated by averaging across categories. Cronbach’s alpha was used to measure inter-question reliability for each category and the general score. Cronbach’s alpha was between 0.7 and 0.8 for all scores except for use of incentives and sanctions (Cronbach’s alpha of 0.54 and 0.69, respectively). To compare scores across categories, the scores were rescaled from 0 to 100 by dividing the average raw score by the maximum values in the distribution within each category and multiplying by 100. We then examined rescaled scores and intervention unit costs using Spearman correlation coefficients.

All data were analyzed using STATA 15 software (Stata IC/15, Stata Corp., College Station, TX, USA) [30] and R Version 3.5.1 [31].

### Ethics approval

The protocol from this study was approved by the Ethics Committee of the National Institute of Public Health, Mexico (CI-1403), the Health Research Ethics Committee of the National Agency for the Control of AIDS, (NACA, FHREC/2016/01/58/08-08-16) and by the Nigerian Institute for Medical Research in Nigeria (NIMR, IRB/17/024). Written informed consent was obtained for all training participants before data collection. No patient data was directly collected, only CBO costs. Salary information was anonymized and therefore individual volunteer/manager consent for salary information was waived by all relevant ethics committees.

## Results

### Demographic characteristics

The CBOs included in the study (n = 31) were spread across 14 states in Nigeria; five were classified in the geographical central/northern region (Kano, Gombe, Abuja, Benue, and Nassarawa), while nine were in the southern region (Anambra, Oyo, Imo, Enugu, Edo, Lagos, Cross Rivers, Rivers, and Akwa Ibom). The CBOs had an average of two funded projects (Table 1) and 498 volunteers working in 2016 on the HIVE intervention, while only an average of 53 worked in HCT. CBOs distributed on average 305,798 male condoms in 2016, though this number varied greatly by CBO (Table 1), while, on average, CBOs distributed considerably fewer female condoms and lubricants when compared to male condoms (5,406 and 7,418, respectively). A greater number of FSWs tested and tested positive in the north/central region than in the southern region (Table 1). More inputs and outputs were observed overall in the SHiPs program. Only one Global Fund site provided HTC services (Table 1).

**Table 1:** Characteristics of a sample of community-based organizations in Nigeria in 2016, overall and by region^a^.

		Region			Program		
	**Overall**	**North/Central**	**South**	**p-value** ^**b**^	**Global Fund**	**SHiPS for MaRPs**	**p-value** ^**b**^
**Total N (%)**	31 (100)	9 (29)	22 (71)		9 (29)	22 (71)	
**Number of projects per CBO mean (SE)**	2.0 (0.2)	1.6 (0.2)	2.2 (0.3)	0.21	2.0 (0.4)	2.0 (0.3)	0.93
**Inputs mean (SE)**							
Number of male condoms distributed	305,798 (75,462)	383,977 (204,867)	273,816 (68,953)	0.52	277,567 (94,495)	374,808 (124,177)	0.57
Number of female condoms distributed	5,406 (2,092)	9437 (6,626)	3593 (689)	0.20	2,321 (856)	6,795 (2,981)	0.33
Number of lubricants distributed	7,418 (2,200)	10,398 (6,024)	6,141 (1,879)	0.38	4,146 (726)	8,820 (3,099)	0.34
Number of volunteers for HCT	53 (12)	80 (34)	43 (9)	0.17	1 (1)	75 (15)	0.003**
Number of volunteers for HIVE	498 (119)	375 (91)	548 (164)	0.52	544 (338)	479 (104)	0.81
**Outputs mean (SE)**							
Number of FSW educated	11,294 (2,239)	12,569 (5,207)	10,773 (2,412)	0.72	3,152 (1,891)	14,625 (2,777)	0.02*
Number of FSW tested	3,326 (685)	5,802 (1,950)	2,265 (363)	0.02*	1,064 (381)	4,295 (888)	0.03*
Number of FSW tested positive	129 (27)	252 (76)	79 (12)	0.002**	52 (15)	161 (35)	0.06^+^
Number of FSW referred for STIs (n = 26)	473 (106)	678 (238)	364 (100)	0.17	62 (23)	624 (129)	0.02*

*** p<0.001

** p<0.01

* p<0.05, + p<0.1

^a^All calculations are for the 2016 fiscal year (January to December)

^**b**^p-values are from two-sample, two-sided t-tests comparing regions

### Total costs and unit costs of HIV prevention services

On average in 2016, the average total cost of providing HIVE services was $69,127, and $29,326 for HCT services (Table 2). The total average cost for STI referrals was $590. When accounting for scale, the average unit cost of HIVE was $22, and similarly, the unit cost of HCT was $19 (Table 2). The average unit cost of STI services was $3 (Table 2). Unit costs across the three interventions vary substantially. The distribution ranged from about $2 to $142 for HIVE, $3 to $127 for HCT, and from $0 to $16 for STI referrals.

**Table 2:** Costs of HIV prevention services in Nigeria in 2016^a,b^.

Prevention service provided	Unit cost					Total cost^c^				
	N	mean	Median	Min	Max	N	mean	Median	Min	Max
**HIV education**	31	21.6	6.3	1.7	141.8	31	69,127	43,937	19,991	371,903
**HIV testing and counselling**	30	18.7	8.1	3.0	126.8	30	29,326	19,011	1,562	100,361
**STI referrals**	26	3.0	1.0	0.2	16.3	26	590	291	11	5114

^a^ All costs estimated for the 2016 fiscal year (January to December)

^b^ All costs are in 2016 US dollars (USD) and reflect the Exchange rate considered: 252.74 NGN per one dollar (2016) average exchange rate, Source of information: http://www.cbn.gov.ng/rates/exchratebycurrency.as

### Unit cost and HIV prevalence by state

**Fig 1:**
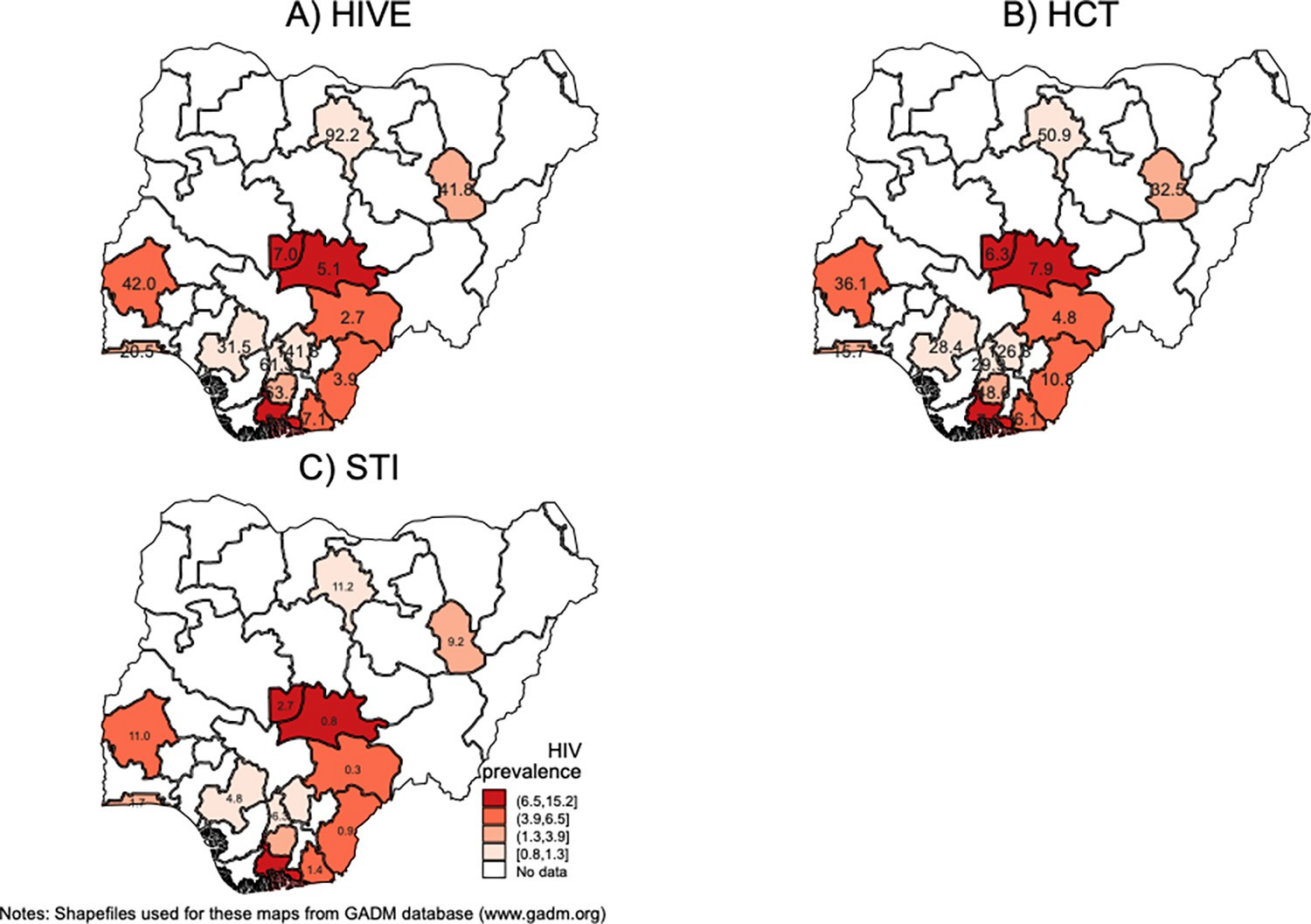
Maps of average unit costs (USD) among CBOs providing HIV prevention services in 2016 and HIV prevalence by Nigerian state for (A) HIV education services, (B) HIV testing and counselling services, and (C) STI treatment referral services. Note: HIV prevalence is depicted in the red scale and shown in percentage. Unit costs are inside the states.

We analyzed unit costs and HIV prevalence across geographical areas and found that across all interventions, Northern states, and Southwest/Southcentral States (Kano, Gombe, Oyo, Lagos Anambra, Imo, Enugu, Edo) had higher unit costs and central and Southeast States (Abuja, Nassarawa, Benue, Rivers, Cross River, Akwa Ibom) had lower unit costs. Average unit costs tended to be relatively lower in states with higher HIV prevalence across all intervention (Fig 1).

### Cost decomposition

**Fig 2:**
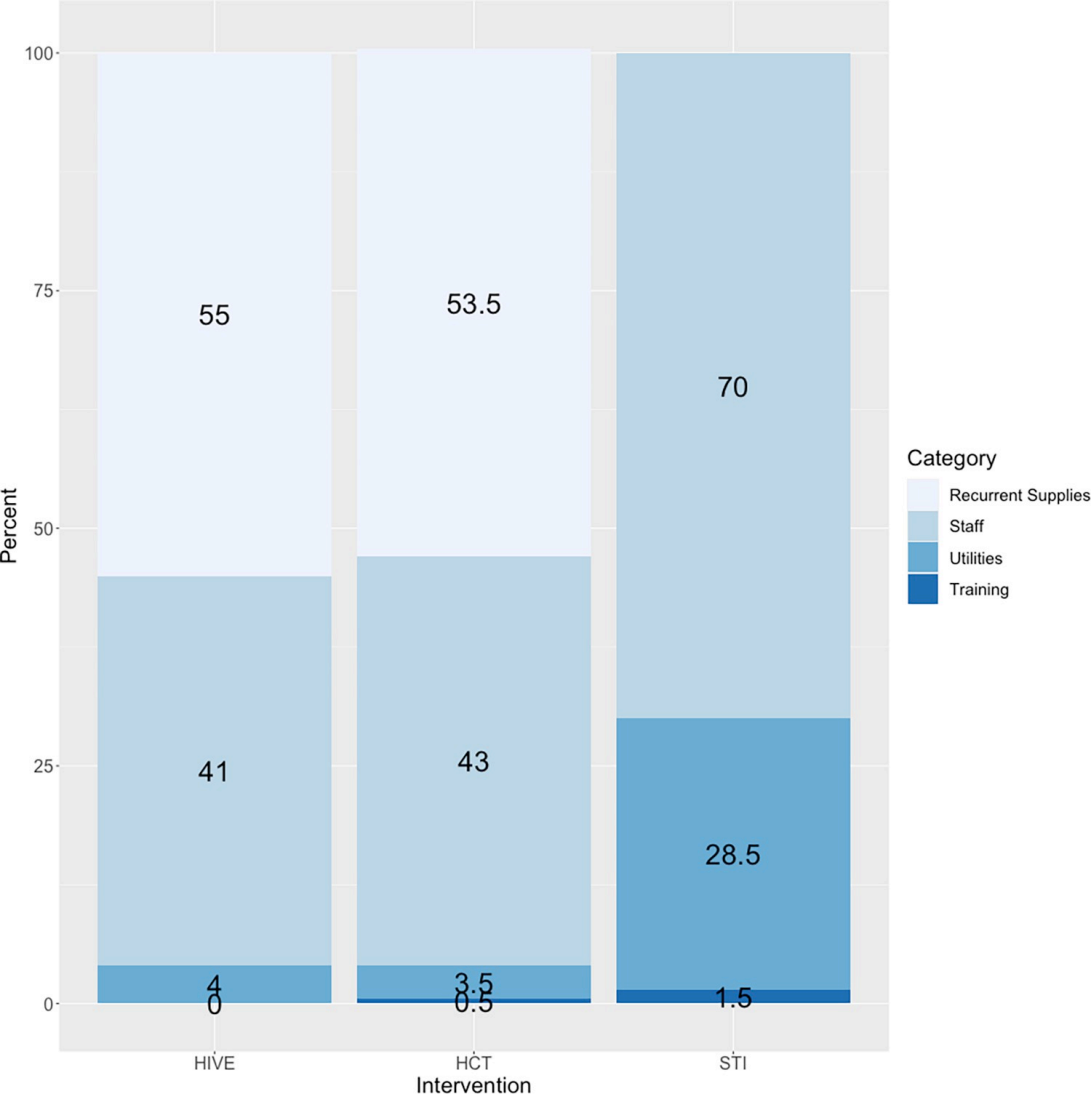
Program costs of a sample of Nigerian community-based organizations by intervention and input type.

To understand the main cost drivers, we decomposed the total cost by input type across the interventions. This exercise allowed us to understand the relevance of each cost input and compare this composition across interventions. The main driver of cost for HIVE was recurrent supplies (55 percent of the total cost), followed by staff salaries (41 percent of the total cost, Fig 2). The relevance of utilities and training was small (5 percent of the HIVE total cost, Fig 2). The cost drivers for HCT services were similar to that of HIVE. In contrast, the main drivers of cost for STI referrals were staff, followed by utilities.

### Scale of services over time

**Fig 3:**
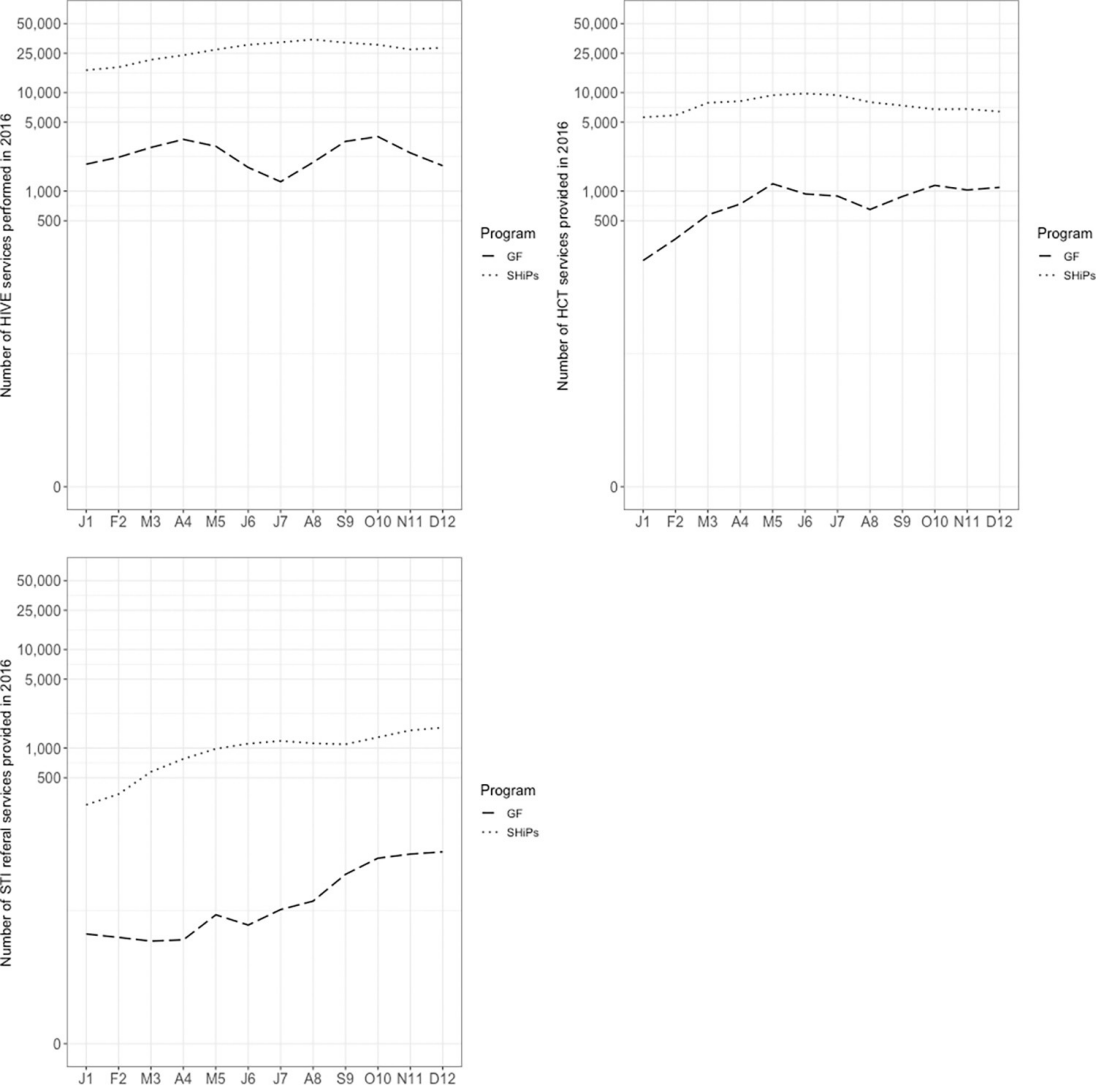
Number of services performed by community-based organizations providing HIV prevention services in Nigeria throughout the 2016 year, by program. Note: the graph shows the rolling 3-month average number of services.

We examined the rolling 3-month average number of services provided over the course of the year for the three interventions according to the program funding (SHIPs vs. Global Fund). We found that the number of women receiving HIV education at SHiPS-funded sites trended upward from January to May and fell toward the end of the year. Global Fund sites fluctuated more, peaking in April and October, and falling in July (Fig 3). The number of women receiving HCT by SHiPS program observed little changes throughout the year, whereas the Global Fund sites had an upward trend. In the case of STI referrals, both sites funded by SHiPS and Global fund had overall ascending trends, with some fluctuation. Services provided for all interventions dropped sharply in July among Global Fund sites.

### Relationship between cost and scale of services

**Fig 4:**
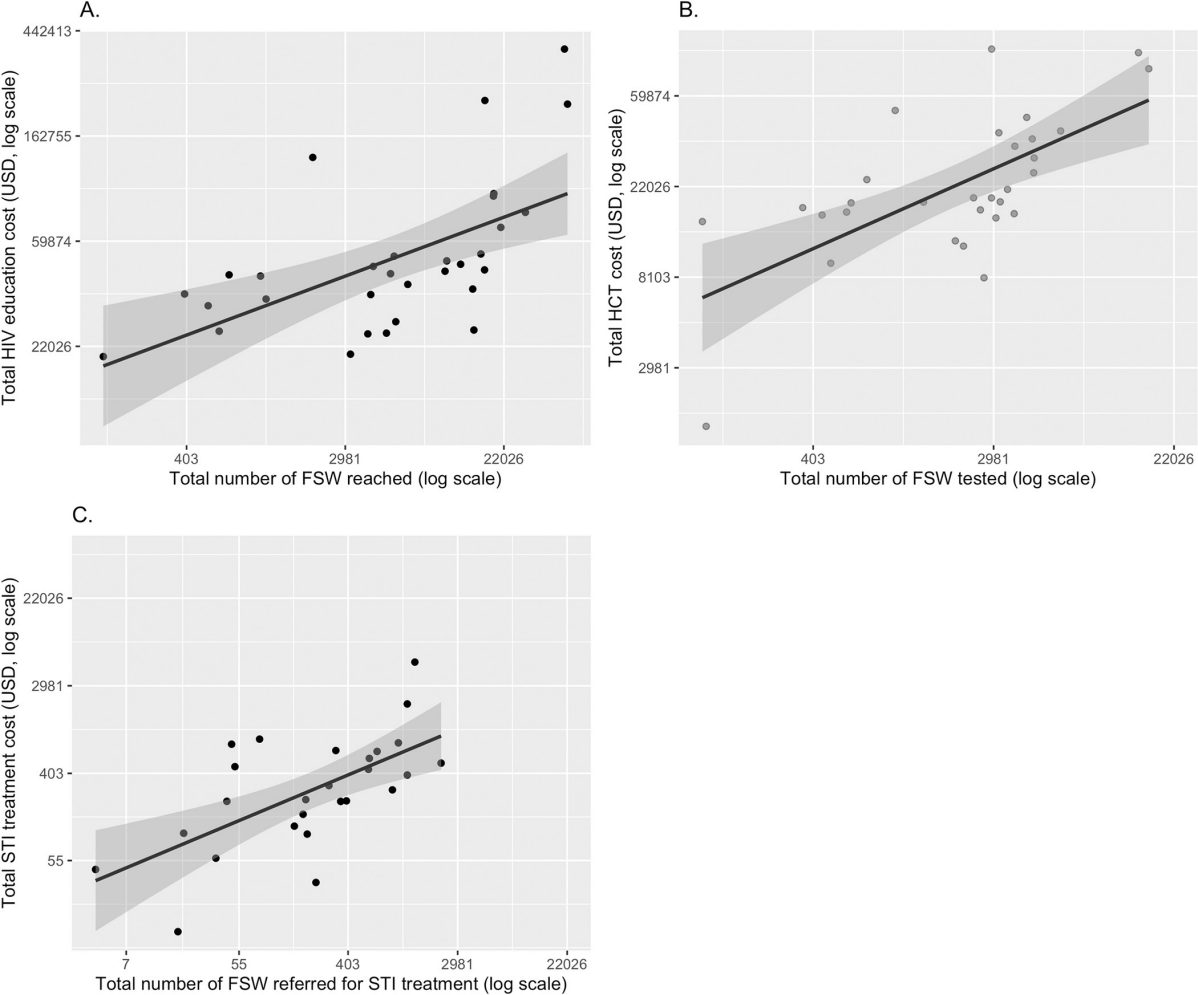
Total cost and scale of services among a sample of Nigerian CBOs providing (A) HIV education services, (B) HIV counselling and testing services, and (C) STI referrals.

**Fig 5:**
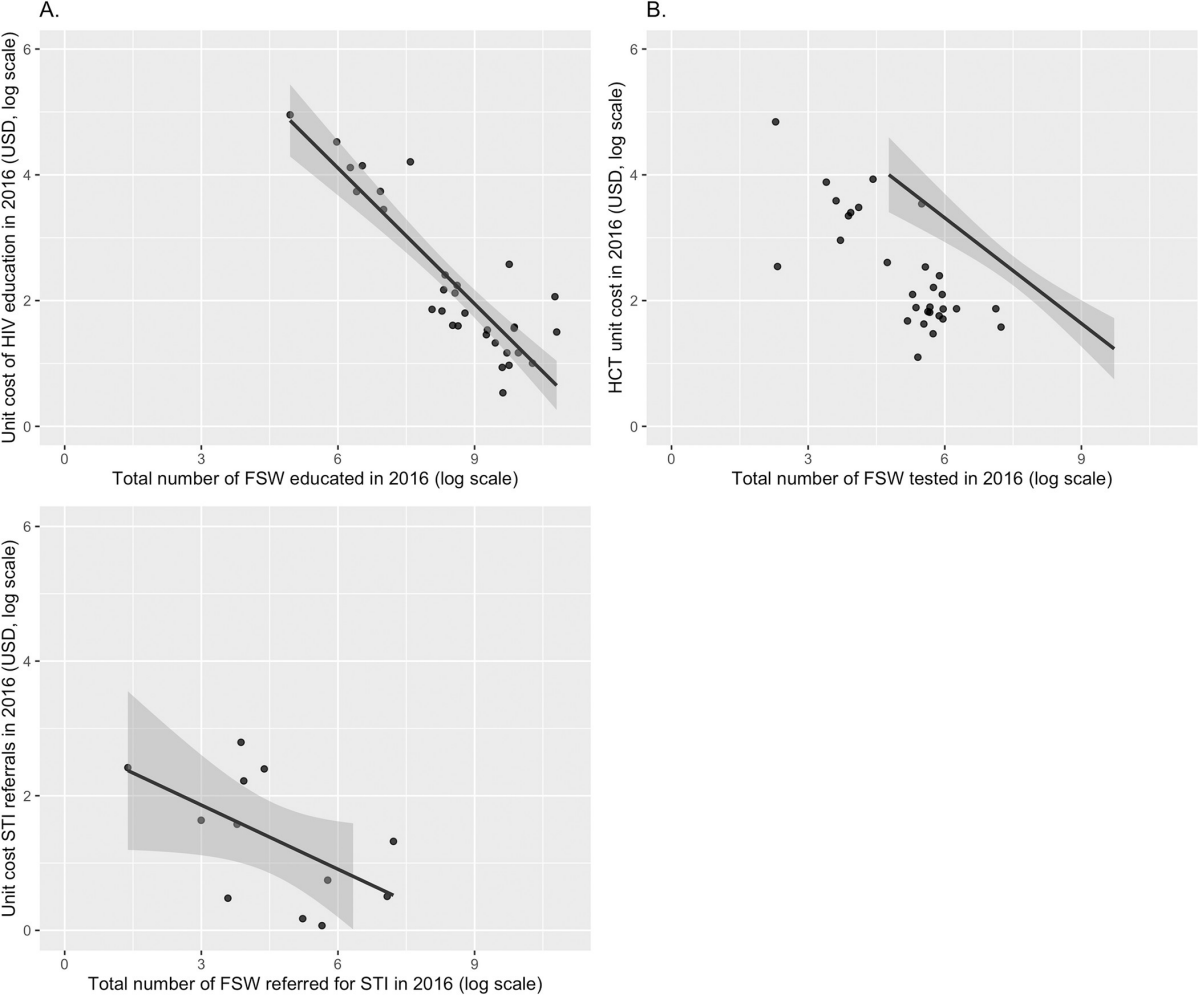
Unit cost and scale of services among a sample of Nigerian CBOs providing (A) HIV education services and (B) HIV counselling and testing services, and (C) STI referrals.

Figs 4 and 5 show the statistical relationship between total and unit costs (y-axes) and the level of service scale (x-axis). The 95 percent confidence intervals for the linear best-fit line are shown in grey along the curves. Total cost and level of scale hold a significant and positive and linear relationship in HIVE and HCT services (Fig 4). There is a significant and negative relationship between unit costs and scale of production, though this negative relationship diminishes as scale grows (Fig 5). Given the distribution of the data, we performed a sensitivity analysis by removing these outliers (defined as greater than 1.25 times the interquartile range) and found the relationships to be similar across the three interventions (results not shown).

Furthermore, we analyzed the relationship between costs unit costs and service scale, through both unadjusted and adjusted OLS log-log regression models. Table 3 shows that unit costs and scale were negatively correlated across all interventions; all the coefficients were statistically significant except for the adjusted STI model. In both the unadjusted and adjusted models, we found evidence of economies of scale. After adjusting for funding program, increasing the annual number of services by 100% was associated with a unit cost reduction of 60, 40 and 10 percent for HIVE, HCT and STI, respectively. Service scale explained 77, 53 and 33 percent of the variation in unit costs for HIVE, HCT and STI services, respectively. The funding program was relevant for all interventions, and those sites funded by SHiPs observed a lower unit cost, when compared to those sites funded by the GF, particularly for the STI intervention. Finally, with regards to the constant term that represents the average fixed costs, Table 3 shows that these costs are higher for HIVE services.

**Table 3:** Relationship between costs and service scale.

	HIVE		HCT		STI	
	**(1)**	**(2)**	**(1)**	**(2)**	**(1)**	**(2)**
	**Unit Cost (ln)**	**Unit Cost (ln)**	**Unit Cost (ln)**	**Unit Cost (ln)**	**Unit Cost (ln)**	**Unit Cost (ln)**
**Service Scale(ln)**	-0.7***	-0.6***	-0.6***	-0.4***	-0.5**	-0.1
	(0.1)	(0.1)	(0.1)	(0.1)	(0.1)	(0.1)
**Program**		-0.7*		-0.8**		-1.9***
		(0.3)		(0.3)		(0.5)
**Constant**	8.4***	7.6***	6.7***	5.9***	2.8***	2.3***
	(0.6)	(0.7)	(0.7)	(0.7)	(0.7)	(0.6)
**Observations**	31	31	30	30	26	26
**Adjusted R-squared**	0.77	0.80	0.53	0.63	0.33	0.59

^a^ Models (1) unadjusted regression results with service scale

^b^ Models (2) adjusted regression results with service scale—adjusting for funding program: 1 if SHiPS, 0 otherwise.

Note: The coefficients reported are the natural log of costs. Each coefficient is from a different regression. Standard errors in parentheses.

*** p<0.001

** p<0.01

* p<0.05, + p<0.1

### Management practices

**Fig 6:**
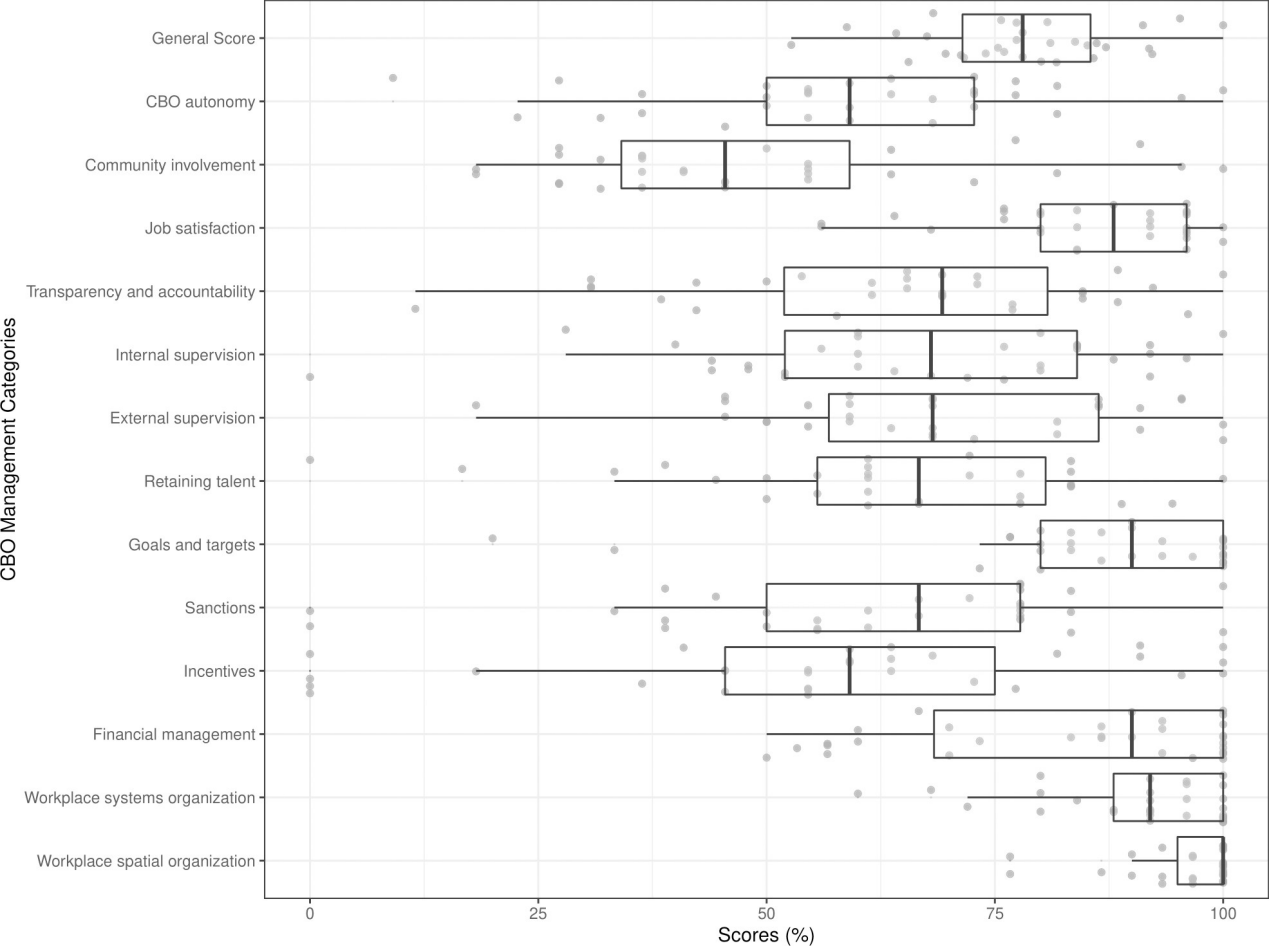
Management scores for community-based organizations providing HIV prevention services in Nigeria in 2016 (n = 31).

The distribution of all the management categories as well as the general management score are shown in Fig 6. The median general management score was 78. There was substantial variation in management practices across the CBOs. Higher scores were meant to reflect ‘better’ management practices. Job satisfaction, goals and targets, financial management, workplace systems organization and workplace spatial organization had median scores above the median general score. Community involvement management had the lowest overall median score. There were CBOs that did not do some practices such as, the use of incentives or sanctions, or did not have a plan in place for retaining talent or doing internal supervision.

**Fig 7:**
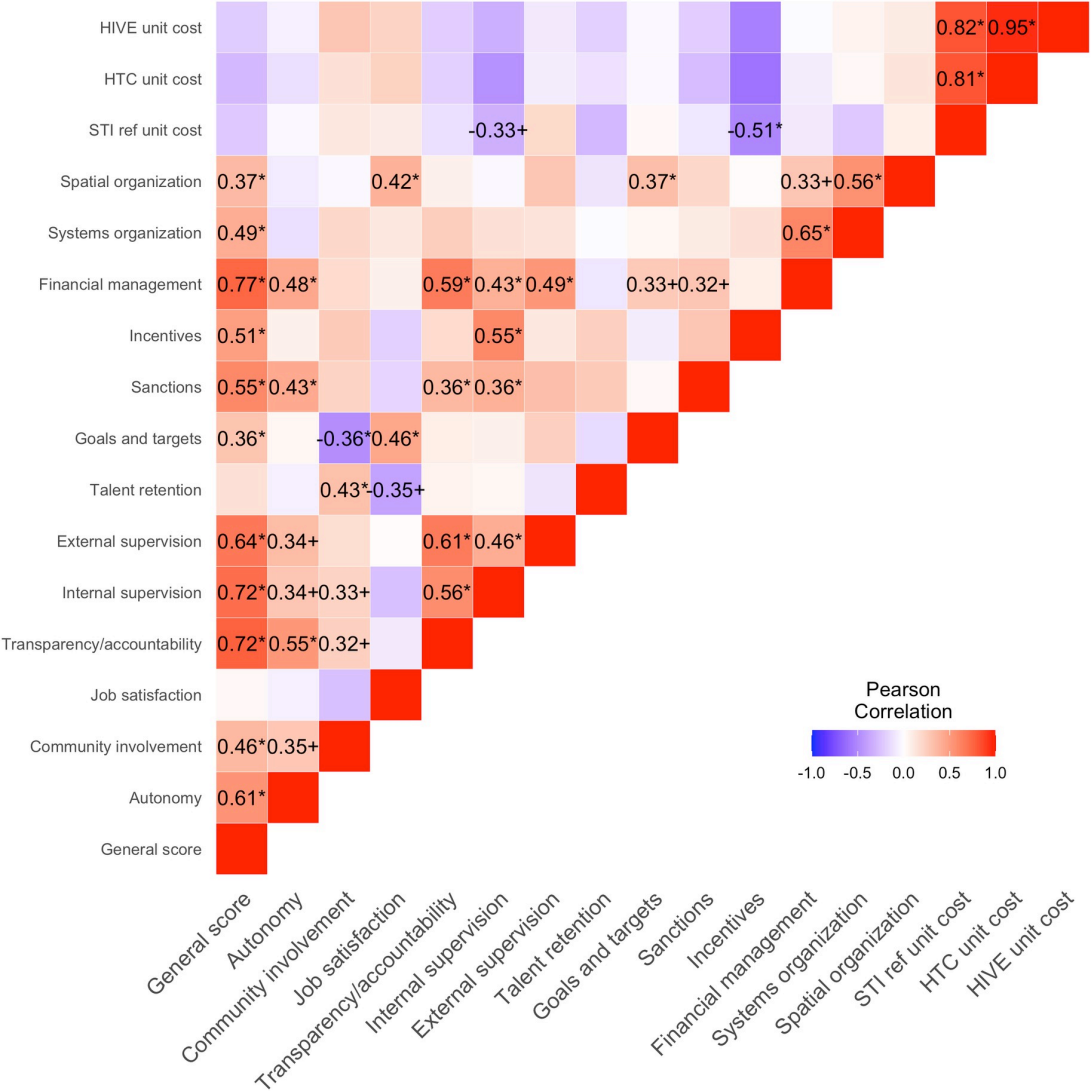
Correlations between unit costs and all management practices among community-based organizations providing HIV prevention services in Nigeria in 2016 (n = 31). Note: * p<0.05, + p<0.1.

We further examined the relationship between management practices using Pearson correlation coefficients (Fig 7). We found no statistically significant correlations between management practices and unit costs, though unit costs and overall management practices are negatively correlated, implying that better management is correlated to lower unit costs. Fig 7 also shows a notable heterogeneity in the direction of the relationships across management practices.

## Discussion

In this study, we have estimated the total and unit costs of three fundamental HIV prevention services for FSW in Nigeria under the provider’s perspective, analyzed the heterogeneity in unit costs across CBOs, and examined potential links between supply-side factors and cost variation. Our estimated costs for HCT services are relatively like a previous study that looked at costs for HCT services among clinics and tertiary health facilities in four sub-Saharan African countries [31]. Average unit costs tended to be relatively lower in states with higher HIV prevalence across all interventions. This is an expected result since it is likely that geographical areas with a higher HIV prevalence will have more people potentially willing to uptake prevention services. If we can assume the state prevalence to be a proxy for service demand, this follows the expected behavior of economies of scale—where demand is higher, unit costs are lower.

When examining drivers of cost, we found that overall, the CBOs spent more financial resources on HIVE than on HCT, but the unit cost per person reached by HIV education was lower (i.e., they reached more people per dollar spent on the intervention). This is likely because educational sessions can easily reach larger groups of people without the additional costs of supplies like test kits. The two main drivers of the costs for HIVE and HCT were recurrent supplies and staff costs. More than 50 percent of the costs are driven by recurrent supplies is likely due to the economic approach used and the particularities of the programs. As stated above, the cost estimation included non-monetary incentives (soap, toilet paper, toothpaste, playing cards, etc.) that are bought by the CBOs to incentivize FSWs to be intervened. We considered these quantities and estimated the costs incorporating the market price for each item. The second main driver of the costs is staff, in this respect our study agrees with other studies on the topic [14, 16]. We found that HIVE and HCT services spent more resources on supplies, whereas STI referrals spent more resources on staff. This might be because HIVE and HCT services need substantial supplies (test kits, condoms, non-monetary incentives, etc.) to deliver the interventions, whereas STI service referrals can be administered with little to no supplies.

We found some evidence of seasonality of outputs—in particular, services for the HIVE intervention tend to peak in August and September, while HCT services peak in June. This finding differs slightly depending on funding source. This may be because as programs end, they account for previous financial commitments and obligations not fulfilled earlier that year. Time periods of higher cost may be particularly ripe for intervention.

We also found evidence of economies of scale; larger facilities tend to have lower costs per FSW served. This agrees with the general relationship between scale and unit cost in the economics literature, particularly that increasing scale allows cost savings, resulting in a per-service fixed costs decrease. These results are comparable and similar to previous literature [14]. Fig 7 also shows the correlation between management practices, overall all management practices are positively correlated among them, but community involvement and goals and targets show a negative relation, which might imply a tension between involving the community in the governance process of the CBO and its own goals and targets.

### Strengths and limitations

Our study makes three main contributions to the ongoing literature about costing HIV interventions. First, even though there are several studies that have estimated HIV intervention costs in key populations and specially in FSWs [14, 15, 17, 22, 26, 32], most of them have focused on the case study of India. A distinctive feature of our paper is that it uses data from a diverse sample of regions in Nigeria, a key HIV priority country in the sub-Saharan African region. Second, we contribute to the body of literature that employs a bottom-up and micro costing approach—in contrast to top-down methods—to estimate the HIV intervention costs. This allows for accurate enumeration of costs utilized on the ground during service delivery, which is particularly important in costing organizations that are decentralized and community-led. Our study collected data at three levels: the CBO, State-partner agencies and the headquarter-organization level and looked exclusively at on-the-ground implementation costs among the implementing CBO organizations. Our study adopted an economic rather than financial perspective when estimating the HIV intervention costs. As with other studies, the salaries of all types of volunteers were included [22, 26, 33], and non-monetary incentives, such as gifts were also taken into account and valued at the market prices.

However, as with any study, this one has its limitations. The sampling of CBOs favors brothel-based FSWs, and results may prove challenging to externalize to non-brothel-based populations. Also, while we sampled all CBOs funded by Global Fund and SHiPs programs, the study’s sample size was still small. By accurately measuring the costs of HIV prevention transmission services across geographic regions and over time, we can better understand both the strengths and weaknesses of investment approaches that merit further study. Furthermore, accurate cost estimation will continue to aid in the planning and implementation of HIV prevention service delivery in Nigeria in the future.

## Conclusion

This study is the first of its kind to use a micro-costing approach to explore cost variation among the FSW population in Nigeria. The considerable heterogeneity of cost and service scale suggests that factors such as month of operation and geographic region may be important to consider in fund allocation. We also found evidence of economies of scale, suggesting that larger CBOs may be better able to reduce overhead costs than smaller CBOs. While supply-side factors such as management were not significantly correlated with cost, more research may be needed to further explore potential levers to improve service delivery efficiency.

## Supporting information

S1 Table Inputs used in each intervention. (DOCX)

S2 Table Definitions of management variables. (DOCX)

S1 File Inclusivity in global research. (DOCX)
