# Supplementary material for: Costs of HIV prevention services provided by community-based organizations to female sex workers in Nigeria
Source: PLoS One. 2023 Mar 13;18(3):e0282826. doi: 10.1371/journal.pone.0282826 (PMC10010541; doi:10.1371/journal.pone.0282826)
Supplement: S1 Table — (DOCX) [file pone.0282826.s001.docx]

**Supplemental Materials S1. Inputs used in each intervention**

| **Inputs** | **Examples** | **Used in which intervention** |
| --- | --- | --- |
| ***Recurrent Supplies*** | HIV Test Kits | HIVE and HTC. Outputs-based weights were used to allocate a cost to each intervention |
|  | Male and Female Condoms |  |
|  | Lubricants |  |
|  | Playing Cards | HIVE |
|  | Key Chains |  |
|  | Educational Posters, Flyers and Brochures |  |
|  | Soap |  |
|  | Tooth Paste and Brush |  |
|  | Tote Bags |  |
| ***Volunteers*** | Counselor Tester | HCT |
|  | Case Management Officers |  |
|  | Venue Outreach Supervisor |  |
|  | Peer Educators | HIVE |
|  | Community Supervisor |  |
|  | Interpersonal Communication Agent |  |
|  | Referral officer | STI |
| ***Staff*** | Executive Director | All, and outputs-based weights were used to allocate a cost to each intervention |
|  | Program Officer |  |
|  | Monitoring and Evaluation Officer |  |
|  | Finance Officer |  |
|  | Data Management Officer |  |
|  | Receptionist |  |
| ***Rent and Utilities*** | Venue rent | All, and outputs-based weights were used to allocate a cost to each intervention |
|  | Electricity |  |
|  | Water |  |
|  | Indoor and Mobile Phone Services |  |
|  | Maintenance |  |
|  | Internet Services |  |
|  | Transportation and Fuel |  |
| ***Training Activities*** | Traning activities related with the implementation of services | All, and outputs-based weights were used to allocate a cost to each intervention |
|  | Training activities related to management or financial practices |  |
|  | Training activities related to better monitoring and evaluation practices |  |
| Notes: "All" means the three interventions (HCT, HIVE and STI) | | |
