# Supplementary material for: Costs of HIV prevention services provided by community-based organizations to female sex workers in Nigeria
Source: PLoS One. 2023 Mar 13;18(3):e0282826. doi: 10.1371/journal.pone.0282826 (PMC10010541; doi:10.1371/journal.pone.0282826)
Supplement: S2 Table — (DOCX) [file pone.0282826.s002.docx]

**Supplementary Material S2. Definitions of management variables**

| **Variable name** | **Questions** | **Cronbach’s alpha** |
| --- | --- | --- |
| **Workplace spatial organization** | - The CBO gets cleaned daily. - Most of the staff organized their workplace every week. - The CBO has a dedicated space for supplies storage. - The supplies for HIV interventions are stored and organized as soon as they arrive. - There is a working system for storing supplies. - The layout of the CBO is organized and easy to navigate | 0.74 |
| **Workplace systems organization** | - The CBO is required to report stock levels, consumption, etc. - Files at the CBO are labeled and organized. - Procedures for staff are documented in a manual. - Procedures are posted on the walls or blackboards of the CBO. - Reminders are posted in a special place of the CBO | 0.73 |
| **Financial Management** | - The CBO provides an HIV financial performance report every month. - The CBO does a financial audit for itself every year on total facilities revenues and expenditures. - Someone outside of the CBO ("externals") come to do a financial audit every year on total CBO revenues and expenditures. - The CBO elaborates spending plans (budget) each year. - The CBO holds meetings to make follow-ups to the spending plan. - The CBO holds meetings to discuss management and administrative issues related to HIV programs or services | 0.77 |
| **Incentives** | If a staff member performs well, does the CBO provide incentives/rewards for good performance, in the form of...   - Time off - Verbal or written recognitions/certificates - Monetary bonuses - Trainings - Preferred schedule - Commodities (e.g., Food basket) | 0.54 |
| **Sanctions** | If a staff member performs poorly, does the CBO apply sanctions for poor performance in the form of... (In case of no sanctions, please select "N/A")   - Verbal or written warnings - Forced leave or relocation - Pay reduction - Less desirable Schedule   Termination | 0.69 |
| **Goals and targets** | The following questions refer to the goals and targets at the CBO.  This varies greatly depending on CBO structure, and some questions may not apply to your CBO.  Please mark N/A if they do not (eg. There is no governing board).   - The CBO sets goals for staff members. - The goals are clearly presented and discussed with all the staff. - The goals are talked about in staff meetings. - A timeline is made to reach the staff goals/targets. - The CBO has goals related to the spending plan (budget) | 0.81 |
| **Retaining talent** | In the following questions, we are referring to staff members that preform their job exceptionally well, going above what is considered “normal” performance.  This is different for each CBO ’s dynamic and may not necessarily apply to your CBO.  If these, they do not (eg. you do not distinguish between different performances of staff), please mark “N/A”.   - How often do high-performing staff leave for improved opportunities at other facilities? - How often are high-performing staff members retained for more than 5 years? - How often are high-performing staff members promoted? - How often are high-performing staff members given an extended contract? - The CBO has goals related to community involvement/interaction. | 0.75 |
| **External supervision received** | Does the external entity valuate the CBO in terms of….   - Stock of supplies - Personnel performance - Budget and expenditure - Quality of service   CBO layout, transportation, etc. | 0.81 |
| **Internal monitoring and review** | Does the CBO engage in periodic internal review or evaluation of...   - CBO stock of supplies - Personnel performance - Budget and expenditures - Quality of service   CBO layout, transportation, etc | 0.74 |
| **Transparency and accountability** | The following questions refer to the interactions the CBO has with external entities, like the community, or external governing bodies.  This can come in many forms and may not always apply to your CBO.  Please mark N/A if this does not (e.g., there is no governing board).   - Does the CBO inform external entities about the performance of staff? - Are meetings organized at the CBO to report CBO performance internally? - Does the CBO inform external entities about their performance on meeting supply targets? - Does the CBO inform external entities about their performance on meeting budget and expenditures? - Are CBO procedures detailed in written form? - Are CBO procedures sent/shown to an external entity? | 0.79 |
| **Autonomy** | CBO autonomy  The following questions refer to the CBO autonomy. Please mark NA if the following assertions does not apply to your CBO.   - How often are decisions about supplies made by the CBO manager (compared to external entities like the Ministry)? - How often does the CBO choose the people that it hires? - How often does the CBO choose the people that it fires? - How often does the CBO choose the number of employees to employ? - How often does the CBO management have the authority to make decisions related to how the budget is spent? - How often does the CBO make decisions about the layout of the CBO? | 0.77 |
| **Community involvement** | The following questions refer to community participation in CBO services. This can come in many forms and may not always apply to your CBO. Please mark N/A if this does not apply to your CBO (e.g., there is no governing board).   - How often does the CBO inform the community about the performance of CBO services? - How often does the governing board meet? - Are community members involved in the governing board? - Does the community have a role in monitoring and providing feedback on the performance of the CBO? - Does the community participate in the budget decision-making process? - Does the community participate in the expenditure’s decisions? | 0.76 |
| **Job satisfaction** | What is your degree of satisfaction relative to:   - Your job position (workload, responsibilities, etc.) - The support you receive from your supervisor. - The social environment (i.e., your relationships with your colleagues) - The opportunities for further career advancement - Your salary | 0.79 |
